# **ω**-3 PUFAs in the Prevention and Cure of Inflammatory, Degenerative, and Neoplastic Diseases

**DOI:** 10.1155/2013/905986

**Published:** 2013-09-16

**Authors:** Achille Cittadini, Gabriella Calviello, Hui-Min Su, Karsten Weylandt

**Affiliations:** ^1^Institute of General Pathology, School of Medicine, Catholic University, 00168 Rome, Italy; ^2^Department of Physiology, National Taiwan University College of Medicine, Taipei 100, Taiwan; ^3^Department of Gastroenterology, Hepatology and Endocrinology and Experimental and Clinical Research Center (ECRC), Charité University Medicine, 13353 Berlin, Germany

The possibility of health benefits associated with dietary omega-3 polyunsaturated fatty acids (*ω*-3 PUFAs) has been described for several chronic conditions, including cardiovascular, neurodegenerative, and neoplastic diseases. A large body of evidence has emerged over the past years to show the critical role played by inflammation in the pathogenesis of these diseases, previously not considered inflammation related. Therefore, it has been recently hypothesized that *ω*-3 PUFAs' effects may be related, at least in part, to their direct anti-inflammatory activity as well as to that of their oxygenated metabolites (17-HDHA, 18-HEPE, resolvins, and protectins). In this special issue G. Calviello and collaborators summarize and comprehensively discuss the current knowledge regarding the modulating effects of *ω*-3 PUFAs on the production of inflammatory cytokines and proresolving or protective lipid mediators in the context of inflammatory, metabolic, neurodegenerative, and neoplastic diseases. 

S. M. Lee and W. S. An present a review on the beneficial effects of *ω*-3 PUFAs on cardiovascular diseases and possible cardioprotective mechanisms of *ω*-3 PUFAs in chronic kidney disease (CKD) patients. They highlight their ability to reduce inflammation, decrease oxidative stress, inhibit platelet activity, exert antiarrhythmic effects, and improve triglyceride levels, both in the general population and in CKD patients. 

Moreover, a research article by B. Parada et al. is presented showing that *ω*-3 PUFAs inhibit the development of premalignant and malignant lesions in a rat model of bladder cancer, possibly not only through their anti-inflammatory properties, but also by their ability to inhibit oxidative stress, proliferation, and angiogenesis. 

It is known that local inflammation plays a central role in the development of colon cancer, and it has been suggested that its suppression may prevent the development of this malignancy. However, the role of nonsteroidal anti-inflammatory drugs (NSAID) in colitis is controversial, and aggravation of disease due to these compounds has been described. T. Köhnke et al. in their paper use the murine dextran-sodium-sulfate- (DSS-) induced colitis model and demonstrate that the treatment of animals with acetylsalicylic acid (ASA) decreases colitis activity and increases the formation of *ω*-3 PUFA-derived lipid mediators with high anti-inflammatory potential, such as 17-hydroxy docosahexaenoic acid (17-HDHA), a docosahexaenoic (DHA, 22:6 *ω*-3) oxygenated metabolite. This finding suggests that the metabolic transformation of DHA incorporated in colon cells in the presence of ASA could, at least in part, explain the colon cancer preventive activity of this drug, and it might argue for a synergistic effect of ASA and *ω*-3 PUFA in the prevention of colon cancer. 

The hypothesis that dietary treatment with *ω*-3 PUFAs may potentiate the effect of antineoplastic drugs has been comprehensively examined by N. Merendino et al. Their review is focused on the current knowledge supporting the potential use of DHA as an adjuvant in tumor chemotherapy, since it has been reported that this fatty acid may enhance the uptake of anticancer drugs, regulate the oxidative status of tumor cells, and inhibit tumor cell invasion and metastasis. 

Another research article is presented by S. Shin et al., showing that DHA induces simultaneously apoptosis and autophagy in prostate cancer cells expressing mutant p53. The authors find that DHA exerts such antineoplastic effects by inducing the generation of mitochondrial reactive oxygen species (ROS) and the ROS-mediated Akt-mTOR signaling.

Several other biological and molecular mechanisms have been so far described to explain the anticancer effects of *ω*-3 PUFAs in prostate cancer. A detailed overview of the possible mechanisms so far hypothesized is presented by Z. Gu and colleagues. The authors report that a large body of preclinical experimental work has demonstrated the high sensitivity of prostate cancer to the protective action of *ω*-3 PUFAs, while in contrast it has not been definitely established whether the ingestion of increased levels of these fatty acids may actually have a protective effect in prostate cancer patients. This represents a highly controversial issue, as outcomes of the epidemiological studies in patients bearing this and other kinds of cancer appear to be conflicting. Several reasons can explain these divergences, and these are discussed here by Z. Gu et al. 

One possible reason for discrepancies in *ω*-3 PUFA studies, observed also among the interventional trials performed in cardiovascular patients, is highlighted by B. B. Albert et al. in this special issue. This paper discusses the possibility that the easy oxidation of the supplemented *ω*-3 PUFAs to lipid peroxides and other secondary oxidation products may make them ineffective or even harmful. The authors therefore recommend that all clinical trials investigating *ω*-3 PUFA harms or benefits report the results of simple assays determining the oxidative state of an oil. This recommendation appears appropriate also in view of the numerous observations [1] suggesting that very high doses of *ω*-3 PUFAs ingested by “high consumers” of fish and leading to very high incorporation of these fatty acids in plasma lipids, may result in high and harmful tissue levels of oxidized *ω*-3 PUFAs. This may result in a lack of health benefits or even in the induction of carcinogenic effects, such as those observed recently in some epidemiological studies. 

This special issue, besides underlining the importance of identifying and ingesting an appropriate amount of nonoxidized *ω*-3 PUFAs, also drives attention to the importance of an optimal dietary balance between *ω*-3 and *ω*-6 PUFAs, that leads to an appropriate *ω*-6/*ω*-3 PUFA ratio in our tissues. The review by E. Murru et al. discusses the results that have so far demonstrated the relationship existing between the *ω*-6/*ω*-3 PUFA ratio present in tissues and the dietary form of these fatty acids. They focus on the findings that have so far shown a more efficient incorporation of eicosapentaenoic acid (EPA) and DHA into tissue phospholipids (PL), when they are mainly esterified to PL already in the diet. 

Finally, the importance of establishing an appropriate dose of dietary *ω*-3 PUFAs, depending on the form of fatty acid supplementation and on the age and conditions of the subjects, is emphasized in the research article by J. Luo et al. They find that the same fish-oil-enriched diet given to dams breeding piglets or to weaning piglet causes a positive effect only on the growth of piglets breast-fed by the fish-oil-supplemented dams. That effect has been related to a reduced tissue production of proinflammatory cytokines. On the contrary, the piglets directly supplemented with fish oil show reduced growth and higher tissue levels of inflammatory cytokines.

On the whole, the papers contained in this special issue aim to examine the beneficial effects of *ω*-3 PUFAs in several chronic diseases and physiological conditions as well as to identify and examine the molecular mechanisms underlying these effects. Moreover, they highlight the importance of establishing and using in the studies a proper *ω*-3 PUFA supplementation, in terms of amount (daily dose), maximal content of oxidative products, and binding of *ω*-3 PUFAs to PL or triglycerides.

*Achille Cittadini* *Gabriella Calviello* *Hui-Min Su* *Karsten Weylandt*
